# Analysis of clinical characteristics and risk factors of infectious mononucleosis in children with splenomegaly

**DOI:** 10.3389/fped.2025.1649348

**Published:** 2025-12-15

**Authors:** YaSong Gao, FangQi Hu

**Affiliations:** Department of Pediatrics, Anqing Municipal Hospital, Anqing, Anhui, China

**Keywords:** infectious mononucleosis, hepatomegaly, splenomegaly, risk factors, clinical characteristics, children

## Abstract

**Background:**

The initial Epstein–Barr virus infection in children often presents as infectious mononucleosis (IM). However, the risk factors for splenomegaly in IM remain unclear. This study investigated the clinical features and risk factors for splenomegaly in children with IM.

**Methods:**

This retrospective study enrolled children diagnosed with IM between 2019 and 2024. Participants were divided into splenomegaly and non-splenomegaly groups. The relevant clinical data and laboratory examination results of the two groups were compared. Furthermore, multivariate logistic regression and receiver operating characteristic (ROC) curve analyses were performed on the relevant variables.

**Results:**

Splenomegaly, a common clinical manifestation, was observed in 43.6% of the 179 children with IM. The splenomegaly group exhibited higher serum levels of alanine aminotransferase, r-glutamyl transpeptidase, and lactate dehydrogenase, along with higher rates of hepatomegaly and eyelid edema (*P* < 0.05). Multivariate analysis identified older age [odds ratio (OR) = 1.333], eyelid edema (OR = 3.242), and hepatomegaly (OR = 22.072) as independent risk factors (*P* < 0.05). ROC curve analysis determined 3.5 years as the optimal cut-off age for predicting splenomegaly.

**Conclusions:**

Older age, hepatomegaly, and eyelid edema are risk factors for splenomegaly in children with IM, with the risk increasing beyond the cut-off age of 3.5 years. These findings provide clinicians with a practical tool to identify children at higher risk and to offer advice on limiting activities to avoid potential complications, especially in settings where advanced imaging is not readily available.

## 1 Introduction

Infectious mononucleosis (IM), caused by the Epstein–Barr virus (EBV), is a proliferative infectious disease affecting the monocyte–macrophage system. The main clinical manifestations include fever, lymphadenopathy, and pharyngitis. In addition to this triad, other symptoms like fatigue, nausea, vomiting, abdominal pain, myalgia, limb swelling, liver enlargement, splenic enlargement, rash, and bilateral eyelid painless swelling can also occur (1–4). The younger a person is, the milder the symptoms tend to be, and these symptoms are usually atypical (5). Primary EBV infection is asymptomatic in most people; however, when the body has a strong immune response, it will present corresponding clinical manifestations, called IM (6, 7). Severe immunocompromised individuals infected with EBV may develop lymphoproliferative diseases or malignant tumors (8). In developing countries, EBV is more common among infants and young children, while in developed countries, it tends to be more common in older children and adolescents (9). IM is a common cause of splenomegaly in adolescents and young adults in the United States (10, 11). Although IM is generally a self-limited disease, with symptoms resolving within a few weeks after infection, some children may still develop severe complications, such as chronic active EBV infection (CAEBV), EBV-associated hemophagocytic lymphohistiocytosis (EBV-HLH), and nasopharyngeal carcinoma. In rare cases, complications of IM can be fatal, with neurological complications, splenic rupture, and upper airway obstruction being the most common causes of IM death (12, 13). Splenic rupture is a rare complication in clinical practice with an incidence rate of 0.1%–0.5% (14). Most splenic ruptures occur spontaneously and without contact. However, any trauma may lead to the risk of splenic rupture, with 1 in every 200 cases of IM complicated by splenic rupture (10). Splenic rupture primarily occurs in individuals aged 15–30 years (27). Therefore, relevant literature typically advises against vigorous activity and contact sports for the first 4–6 weeks following the onset of symptoms (13). However, due to their extensive range of activities, the incidence of accidental injuries in children is significantly higher than that in adults. Literature reports indicate that splenic rupture in IM occurs only in patients with splenomegaly (15). Therefore, we need to be highly vigilant about the risk of splenic rupture in children with splenomegaly. To the best of our knowledge, no prior studies have examined IM in combination with splenic enlargement. In view of this gap, we conducted this study.

## 2 Materials and methods

### 2.1 Subjects and methods

#### 2.1.1 General information

Children admitted to the Department of Pediatrics at Anqing Municipal Hospital, Anhui Province, China, and diagnosed with IM between January 2019 and September 2024 were enrolled. This study was approved by the Ethics Committee of Anqing Municipal Hospital (Approval Number: 2024-175) and conducted in accordance with the Declaration of Helsinki. Informed written consent was sought from parents or guardians of all the study participants. None of the patients in this study had a documented history of amoxicillin use. The participants were divided into the splenic enlargement group and non-splenic enlargement group according to the presence or absence of splenic enlargement in the ultrasound results. All children diagnosed with IM underwent color Doppler ultrasound examination upon admission. For those patients identified with splenomegaly, a follow-up ultrasound was performed prior to discharge. If splenomegaly persisted at discharge, a subsequent ultrasound was scheduled at the 1-month outpatient follow-up. Telephone follow-up at 3 months confirmed that splenic size had returned to normal in all these cases.

Inclusion criteria: (1) The age of the enrollment was <14 years; (2): (a) Clinical diagnosis case: the participants were required to meet any three of the following clinical manifestations or any one of the following laboratory tests C or D. (b) Confirmed case: The participants were required to meet any three of the following clinical manifestations and any one of laboratory tests A or B (16, 17). Clinical manifestations: (1) fever, (2) pharyngitis, (3) enlargement of cervical lymph nodes, (4) liver enlargement, (5) splenic enlargement, and (6) edema of eyelids. Laboratory examination: (1) positive for anti-EBV-CA-IgM and anti-EBV-CA-IgG antibodies, with anti-EBV-NA-IgG negative [Enzyme-Linked Immunosorbent Assay (ELISA) Kit (manufactured by EUROIMMUN, Germany)]; (2) single anti-EBV-CA-IgG antibody positive, with EBV-CA-IgG identified as low-affinity antibodies; (3) proportion of atypical lymphocytes in peripheral blood ≥0.10; and (4) the proportion of peripheral blood lymphocytes in children aged 6 and above is >0.50 or the absolute value of lymphocytes is >5.0 × 10^9^/L.

Exclusion criteria: (1) Children with incomplete clinical data were excluded; (2) Children with hematological diseases, malignant neoplastic diseases, immunodeficiency disorders, autoimmune diseases, or with other severe systemic or organic diseases causing liver and kidney function damage were rejected; (3) Patients with cytomegalovirus-induced mononucleosis or other mononucleosis-like infections were excluded. Criteria for liver enlargement: Under ultrasound localization, the liver exceeds the costal margin by 2 cm in children under 3 years, by 1 cm in children aged 3–7 years, and by any margin in children over 7 years. Criteria for splenic enlargement: Under ultrasound, the spleen surpasses the costal margin. These liver and spleen measurements were taken when the children were quiet. Criteria for lymph node enlargement: The short axis of cervical lymph nodes >1 cm was considered enlargement (17).

### 2.2 Observation indicators

Clinical data were obtained by consulting the electronic medical record system of the hospital and recording the basic information of the children, such as gender, age, and duration of hospitalization. The laboratory indicators on admission included lymphocyte count, monocyte count, alanine aminotransferase (ALT), r-glutamyl transpeptidase (r-GT), lactate dehydrogenase (LDH), C-reactive protein (CRP), and percentage of atypical lymphocytes in peripheral blood. Ultrasound findings included the status of liver and spleen after admission. Clinical manifestations included duration of fever, maximum temperature, size of cervical lymph nodes, pharyngitis, and eyelid edema. Downey cells were identified on microscopic examination of the peripheral blood smear.

### 2.3 Statistical analysis

Continuous variables were expressed as mean ± standard deviation (M ± SD) for variables conforming to a normal distribution and as median (interquartile range) for variables not normally distributed. An independent sample *T*-test or Mann–Whitney *U*-test was used. Categorical variables were expressed as frequencies (%), and comparisons among groups were made by using the chi-square test or Fisher's exact probability method. Logistic regression analysis was used to explore the risk factors for splenomegaly, Receiver operating characteristic (ROC) curve analysis was used to analyze the area under the curve (AUC) of age and calculate the best cut-off value, sensitivity, and specificity. All statistical analyses were conducted using IBM SPSS (Version 26.0). A *P-*value <0.05 was considered statistically significant.

## 3 Results

### 3.1 Characteristics of study population

During the retrospective investigation, data were collected from 179 children who met the diagnostic criteria for IM. They were categorized into a splenomegaly group (*n* = 78) and a non-splenomegaly group (*n* = 101). The cohort consisted of 99 males (55.3%) and 80 females (44.7%), with a male-to-female ratio of 1.24:1. Among these children, 168 were confirmed cases and 11 were clinically diagnosed cases. No cases of splenic rupture were observed. The toddler period (1–3 years old) was the most common age of onset for IM, accounting for 37.4%, followed by the preschool period (3 to 6–7 years old) at 30.2%. Enlarged cervical lymph nodes were the most prevalent clinical manifestation, occurring in 159 cases (88.8%), followed closely by fever in 157 cases (88.7%). Pharyngitis was observed in 106 cases (59.2%), and palpebral edema was found in 31.3% of the children (56 cases). In contrast, rash was reported in only 15.1% of the children (27 cases). Concentrations of ALT, r-GT, and LDH were significantly elevated in the splenomegaly group compared with the non-splenomegaly group (*P* < 0.05). There was no statistically significant difference between the two groups in terms of maximum temperature, duration of fever, white blood cell count, lymphocyte count, monocyte count, neutrophil count, and percentage of peripheral blood heterogeneous lymphocytes (*P* > 0.05) (Table 1).

**Table 1:** Demographic, clinical characteristics, and laboratory findings of children with infectious mononucleosis.

Variable	Splenomegaly		*P*
	Yes (*N* = 78)	No (*N* = 101)	
Infant period, *N* (%)	2 (2.60%)	19 (18.80%)	**0**.**000**^a^
Toddler period, *N* (%)	18 (23.10%)	49 (48.50%)	
Preschool period, *N* (%)	33 (42.30%)	21 (20.80%)	
School-age period, *N* (%)	21 (26.90%)	12 (11.90%)	
Adolescence period, *N* (%)	4 (5.10%)	0 (0.00%)	
Gender (M), *N* (%)	44 (44.4%)	55 (55.6%)	0.794
Duration of hospitalization (days)	7 (6–9)	7 (5.5–8)	**0**.**010**
*T*_max_ (°C)	39.00 ± 0.99	38.99 ± 1.09	0.972
Duration of fever (days)	5.56 ± 3.66	4.79 ± 3.20	0.135
Leukocytes (10^9^/L)	10.63 (9.16–14.09)	11.39 (8.03–14.29)	0.424
Lymphocytes (10^9^/L)	7.5 (5.98–10.23)	7 (4.80–9.40)	0.059
Monocytes (10^9^/L)	0.96 (0.58–1.80)	0.95 (0.62–1.78)	0.905
Neutrophils (10^9^/L)	2.15 (1.48–2.93)	2.3 (1.35–3.60)	0.468
ALT (IU/L)	74 (29.50–146.25)	37 (20.5–113.0)	**0**.**014**
Alkaline phosphatase (IU/L)	200.5 (154.75–264.25)	187 (151.50–238.50)	0.220
r-GT (IU/L)	26.5 (15.00–69.25)	15 (11–38)	**0**.**003**
LDH (IU/L)	473.5 (410.25–536.75)	435 (354–536)	**0**.**024**
CRP (mg/L)	5.40 (2.58–10.92)	5.63 (1.74–13.73)	0.888
Proportion of atypical lymphocytes	0.16 ± 0.11	0.14 ± 0.10	0.220
Clinical Manifestation			
Fever, *N* (%)	71 (91.0%)	86 (85.1%)	0.235
Hepatomegaly, *N* (%)	24 (30.8%)	2 (2.0%)	**0**.**000**
Lymphadenectasis, *N* (%)	69 (88.5%)	90 (89.1%)	0.892
Rash, *N* (%)	7 (9.0%)	20 (19.8%)	**0**.**045**
Palpebral edema, *N* (%)	34 (43.6%)	22 (21.8%)	**0**.**002**
Pharyngitis, *N* (%)	54 (69.2%)	52 (51.5%)	**0**.**017**

*T*_max_, maximum temperature; CRP, C-reactive protein; LDH, lactate dehydrogenase; ALT, alanine aminotransferase; r-GT, r-glutamyl transpeptidase. Bold values indicate that the statistical result has a *P* < 0.05.

^a^ Fisher exact probability method.

### 3.2 Risk factors for splenomegaly in children with IM

Multivariate logistic regression analysis was performed on variables that demonstrated statistical significance in the univariate analysis of IM combined with splenic enlargement, including age, duration of hospitalization, lymphocytes, LDH, hepatomegaly, rash, palpebral edema, and pharyngitis. The results indicated that age [odds ratio (OR) = 1.333, 95% CI: 1.142–1.557, *P* = 0.000), eyelid edema (OR = 3.242, 95% CI: 1.479–7.107, *P* = 0.003), and liver enlargement (OR = 22.072, 95% CI: 4.663–104.482, *P* = 0.000) were independent risk factors for IM combined with splenic enlargement (Table 2).

**Table 2:** Univariate and multivariate analysis of risk factors for splenomegaly in children with infectious mononucleosis.

Variable	Univariate		Multivariate	
	OR (95% CI)	*P*	OR (95% CI)	*P*
Age	1.393 (1.216–1.597)	0.000	1.333 (1.142–1.557)	**0**.**000**
Duration of hospitalization	1.171 (1.029–1.333)	0.017	1.141 (0.966–1.348)	0.120
Lymphocytes	1.092 (1.001–1.191)	0.046	1.072 (0.944–1.217)	0.284
ALT	1.003 (1.000–1.006)	0.089		
r-GT	1.004 (0.999–1.009)	0.083		
LDH	1.003 (1.000–1.005)	0.047	0.999 (0.995–1.003)	0.735
Hepatomegaly	22.000 (5.007–96.656)	0.000	22.072 (4.663–104.482)	**0**.**000**
Rash	0.399 (0.159–1.000)	0.050	0.775 (0.232–2.585)	0.678
Palpebral edema	2.775 (1.447–5.319)	0.002	3.242 (1.479–7.107)	**0**.**003**
Pharyngitis	2.120 (1.141–3.938)	0.017	1.239 (0.560–2.745)	0.597

LDH, lactate dehydrogenase; ALT, alanine aminotransferase; r-GT, r-glutamyl transpeptidase. Bold values indicate that the statistical result has a *P* < 0.05.

### 3.3 ROC curve analysis of age pair in predicting IM with splenomegaly

**Figure 1:**
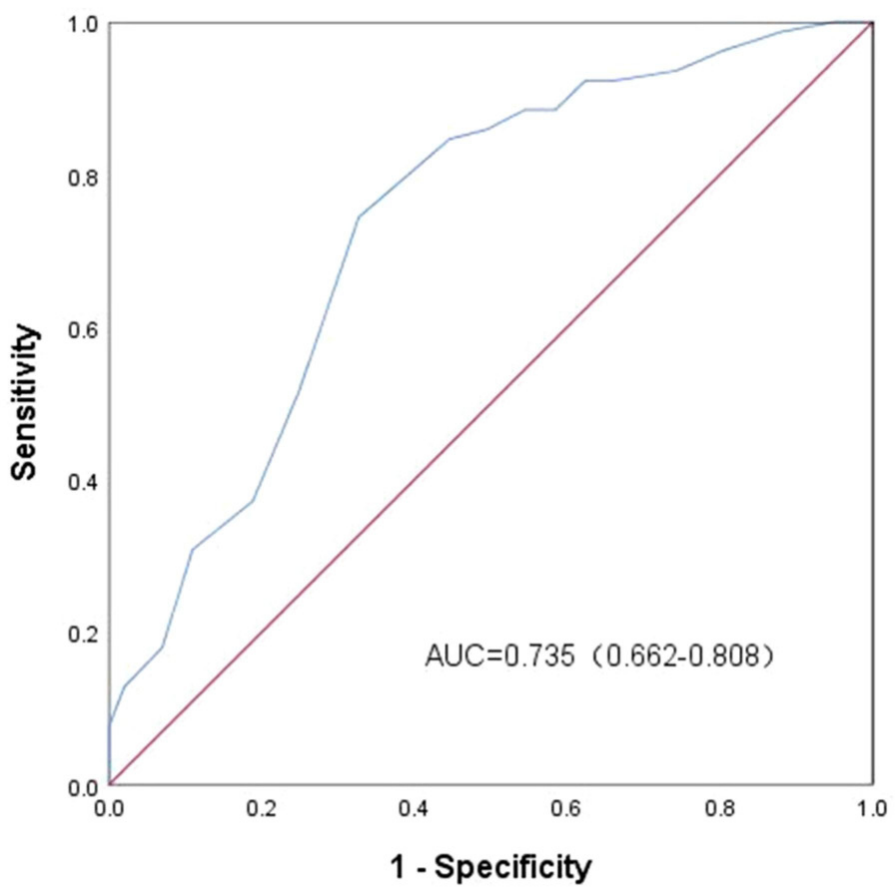
ROC curve analysis of age in predicting infectious mononucleosis with splenomegaly.

ROC curve analysis was performed on age, and the AUC was 0.735. Using 3.5 years as the best cut-off value for IM complicated with splenomegaly, the sensitivity and specificity were 74.4% and 67.3%, respectively (Figure 1).

## 4 Discussion

IM is a common infectious disease caused by the EBV, which is globally prevalent and exhibits a high incidence in both adults and children (7). Splenomegaly is a common clinical manifestation of IM, and in severe cases, it can lead to life-threatening splenic rupture. In this study, we identified age, liver enlargement, and eyelid edema as the most common manifestations of splenomegaly in children with IM. The incidence is higher in boys than in girls, and cervical lymph node enlargement is the most common clinical manifestation. In clinical practice, for older children hospitalized due to EBV infection—or those with liver enlargement, eyelid edema, or all three conditions—clinicians must be vigilant for splenomegaly caused by EBV infection, as it may lead to life-threatening splenic rupture.

The onset of splenomegaly in IM occurs within days, with obvious enlargement during the initial phase. Splenic size peaks within 1–3 weeks and then subsides gradually over 4–6 weeks. Splenomegaly develops in the majority of children with IM, while hepatomegaly is observed in only a minority of cases (18). Previous studies have reported splenomegaly rates of 41%–61.5% and hepatomegaly rates of 10% in IM patients (17, 19). Our findings are consistent with these reports, with splenomegaly present in 43.6% and hepatomegaly in 14.5% of our study cohort. As the dominant lymphoid organ, comprising roughly 99% of lymphoid tissue, the spleen is the largest organ of its kind in the human body; nevertheless, splenomegaly is directly attributed to lymphocyte infiltration within the spleen. However, splenomegaly results from infiltration of the spleen by lymphocytes, with the degree of enlargement correlating with the extent of EBV-infected B lymphocytes, CD8^+^ T cells, and NK cells present in the tissue (20, 21).

The present study demonstrated a male predominance in pediatric IM, with boys accounting for 55.3% of cases and a male-to-female ratio of 1.24:1.The peak onset ages were the toddler (37.4%) and preschool (30.2%) periods, consistent with previous studies (7, 22). While the highest incidence of splenomegaly was observed in preschool children (42.30%), progressively lower rates were seen in school-age and adolescent populations. According to this study, cervical lymph node enlargement was the most frequent clinical finding, followed in frequency by fever. Although children in the splenomegaly group exhibited a numerically higher maximum body temperature, the difference compared to the non-splenomegaly group did not reach statistical significance. In our cohort, rash was observed in merely 15.1% of cases and was predominantly non-specific, corroborating the 5%–15% incidence reported by Ciccarese et al. (23). Patients in the splenomegaly group demonstrated significantly elevated levels of ALT, r-GT, and LDH, along with a higher prevalence of hepatomegaly compared to the non-splenomegaly group. The pathogenesis involves EBV infection of the lymphatic system, which triggers excessive production of inflammatory factors. This cascade accounts for the observed hepatomegaly and peripheral lymphadenopathy. EBV infection triggers the release of inflammatory factors that contribute to hepatic damage (24). Previous studies have established that elevated transaminases, particularly ALT, occur in 40%–80% of pediatric cases. As the primary indicator of hepatocellular injury, ALT is localized predominantly in the cytoplasm of hepatocytes. Patients with hepatosplenomegaly demonstrated higher LDH concentrations than those without, as reported in the literature (25). Our results differ from those of Shao et al. (26), who proposed LDH as a potential prognostic factor in EBV-related hepatosplenic IM, whereas our data do not support this association. The discrepancy may be attributed to the different study populations: Shao et al. focused on EBV-associated hemophagocytic syndrome, whereas our study involved a distinct patient cohort.

Based on the results of the multivariate logistic regression analysis, age (OR = 1.333, 95% CI: 1.142–1.557), eyelid edema (OR = 3.242, 95% CI: 1.479–7.107), and liver enlargement (OR = 22.072, 95% CI: 4.663–104.482) were identified as significant risk factors for splenomegaly in children with IM (OR > 1, *P* < 0.05). Among these factors, liver enlargement was associated with a markedly higher risk compared to age and eyelid edema. Furthermore, ROC curve analysis of age revealed that using 3.5 years as the cut-off value for predicting splenomegaly in children with IM provided moderate diagnostic accuracy, as reflected in its sensitivity and specificity. In view of these findings, it is recommended that high-risk children (those with ≥2 risk factors) undergo strict activity restrictions and regular follow-up ultrasounds, and receive education on splenic rupture. They should be advised to seek urgent medical attention if abdominal pain occurs.

However, our study also had some limitations. First, this was a retrospective, single-center study with a small sample size, and the results may have been influenced by potential confounding factors such as comorbidities. Finally, as a retrospective study, some indicators (such as bilirubin, aspartate transaminase (AST), coagulation function, and lymphocyte subsets) were not available in the cases. Therefore, prospective studies with a larger sample size are required to further verify the relevant results. Future studies should incorporate more comprehensive laboratory indicators and examinations to establish a more accurate and reliable prediction model.

## 5 Conclusions

In conclusion, older age, hepatomegaly, and eyelid edema are risk factors for splenomegaly in children with IM, particularly when the child is older than 3.5 years. This study has enhanced our understanding by providing clinicians with an easily applicable tool to identify children at higher risk and offer guidance on activity restrictions to prevent complications. The simplicity of this risk mode, based on easily assessed clinical parameters, facilitates its use in routine practice, especially in settings where advanced imaging is difficult to obtain.

## Data Availability

The original contributions presented in the study are included in the article/Supplementary Material; further inquiries can be directed to the corresponding author.
